# Study of Bio-Pharmaceutical and Antimicrobial Properties of Pomegranate (*Punica granatum* L.) Leathery Exocarp Extract

**DOI:** 10.3390/plants10010153

**Published:** 2021-01-14

**Authors:** Hazem S. Elshafie, Lucia Caputo, Laura De Martino, Shimaa H. Sakr, Vincenzo De Feo, Ippolito Camele

**Affiliations:** 1 School of Agricultural, Forestry, Food and Environmental Sciences (SAFE), University of Basilicata, Viale dell’Ateneo Lucano 10, 85100 Potenza, Italy; hazem.elshafie@unibas.it (H.S.E.); shimaa.chemist@yahoo.com (S.H.S.); 2 Department of Pharmacy, University of Salerno, 84084 Fisciano, Italy; lcaputo@unisa.it (L.C.); ldemartino@unisa.it (L.D.M.); defeo@unisa.it (V.D.F.); 3 Department of Chemistry, Faculty of Science, Zagazig University, 44511 Zagazig, Egypt

**Keywords:** plant extracts, natural products, biocontrol, plant pathogens, antioxidant, anti-acetylcholinesterase

## Abstract

Pomegranate (*Punica granatum* L.) fruits are important sources of vitamins and minerals and widely used in the dietary supplement industry. An aqueous extract of its leathery exocarp (LEP) was obtained by a solid-phase micro-extraction method. The antifungal activity was examined against the phytopathogenic fungi, *Fusarium oxysporum*, *Phytophthora cinnamomi*, *Penicillium digitatum* and *Botrytis cinerea*, and the antibacterial activity was evaluated against *Escherichia coli*, *Xanthomonas campestris,* *Bacillus megaterium* and *Clavibacter michiganensis*. The antimicrobial assays showed, in some cases, a promising antimicrobial effect compared to the synthetic drugs. The possible anti-acetylcholinesterase and antioxidant activities of the LEP extract were investigated by the Ellman’s assay and 2,2-diphenyl-1-picrylhydrazyl (DPPH) test, respectively, and their results showed that the LEP extract has an effective anti-acetylcholinesterase inhibitory effect and an antioxidant activity. Thus, the LEP extract could be valid as a candidate for further studies on the use of pomegranate in neurodegenerative diseases as a food preservative and a suitable substitute to control several phytopathogens.

## 1. Introduction

Pomegranate (*Punica granatum* L.) is consumed worldwide either as fruit or juice [1,2]. In many countries, the fruit, peel and root of pomegranate are widely used for herbal remedies [3,4]. Fruits are important source of vitamins and minerals and widely used in the dietary supplement industry [5,6], whereas peels account for 50% of the whole fruit and are rich in phenols and flavonoids [7].

Pomegranate fruits are rich in biologically active substances [5,8,9], such as acids, sugars, vitamins, minerals and phenolic compounds, which are considered strong antioxidant agents [10,11]. Hence, pomegranate extracts have several biological properties among them, anti-inflammatory, anticancer and antioxidant [5,6].

Recently, there has been a great interest regarding the role of dietary antioxidants in neurodegenerative diseases, such as Alzheimer’s disease (AD) [12]. Although pomegranate is rich in antioxidant polyphenols and oxidative stress may play a key role in neurodegenerative disease, few in vivo and in vitro studies analyzed its activity as a potential inhibitor of acetylcholinesterase (AChE) [12,13,14]. In AD transgenic mice, a dietary supplementation with 4% of pomegranate attenuated the oxidative damage and reduced the activity of AChE, restoring its normal levels [12]. Furthermore, an ethanol extract of pomegranate leaves or peels showed good AChE inhibition activities [13,14].

Pomegranate fruits also showed a promising antimicrobial effect against some human norovirus infections, such as the influenza virus, due to the effect of the polyphenol fraction of pomegranates [15,16]. In addition, Johann and coworkers [17] reported that a *P. granatum* extract exhibited an effective antifungal activity against *Paracoccidioides brasiliensis*.

The pomegranate leathery exocarp extract (LEP) is rich in hydrolysable tannins and phenolic compounds, which are multiple esters characterized by antioxidant and anti-inflammatory properties [18,19,20], moreover the peel extract has been proposed as a safe antioxidant agent [3,21]. Phenolic compounds having remarkable antimicrobial activity, such as flavonoids and tannins, could be used as natural microbicides against several phytopathogenic microorganisms [22,23,24,25]. In addition, punicalagin isolated from pomegranate peels showed a strong antimicrobial activity against *Candida albicans* and *C. parapsilosis* [26], and the combination of punicalagin and fluconazole showed a synergistic activity. A pomegranate extract was able to inhibit significantly the growth of serious human pathogenic bacteria such as *Clostridia* and *Staphylococcus aureus* [27].

Recently, there has been a growing interest in antioxidant substances as natural preservatives in the food industry and as a coadjutant in the treatment of neurodegenerative diseases [12,28]. On the other hand, there is also a necessity to find out new natural substitutes for chemical antimicrobial agents for different human and phytopathogenic microorganisms [29,30,31,32,33,34,35]. Hence, the main objectives of the current research are to investigate some biopharmaceutical and antimicrobial activities of the pomegranate leathery exocarp (LEP) extract as follows: (i) antibacterial activity against some common human and phytopathogenic bacteria, both Gram-negative bacteria (G−ve) *Escherichia coli* Migula and *Xanthomonas campestris* Pammel and Gram-positive bacteria (G+ve) *Bacillus megaterium* de Bary ITM100 and *Clavibacter michiganensis* corrig. (Smith); (ii) antifungal activity against *Fusarium oxysporum* von Schlechtendal, *Phytophthora cinnamomi* Rands, *Penicillium digitatum* Sacc. and *Botrytis cinerea* Pers; (iii) a possible anti-acetylcholinesterase effect and (iv) a possible antioxidant activity.

## 2. Results

### 2.1. Antibacterial Activity

**Figure 1:**
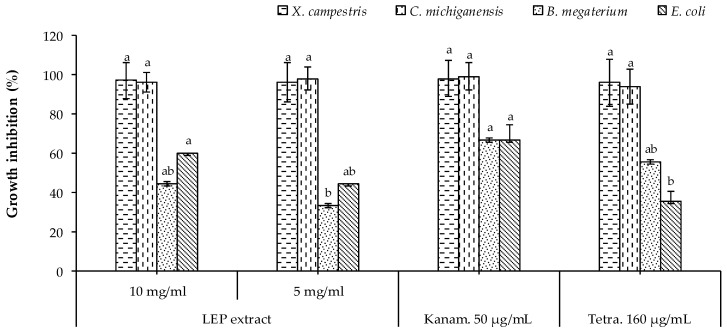
Antibacterial activity of the leathery exocarp (LEP) extract. Bars with different letters indicate mean values significantly different at *p* < 0.05, according to a one-way ANOVA combined with Tukey’s B post-hoc test. Data are expressed as the mean of three replicates ± SD.

The LEP extract significantly inhibited the growth of *X. campestris* and *C. michiganensis* at the two tested concentrations (5 and 10 mg/mL) equal to tetracycline (160 µg/mL) and kanamycin (50 µg/mL), as illustrated in Figure 1. Regarding *B. megaterium*, the higher concentration of the LEP extract (10 mg/mL) showed a promising bactericidal effect slightly lower than kanamycin, whereas the lower tested concentration (5 mg/mL) showed a moderate activity equal to tetracycline (Figure 1). On the hand, only the higher concentration of the extract (10 mg/mL) showed a significant bactericidal effect against *E. coli* equal to kanamycin, whereas the lower concentration of the LEP extract (5 mg/mL) showed also a higher bactericidal activity against *E. coli* than tetracycline (Figure 1). In general, the LEP extract showed promising antibacterial activity compared to the two tested references, mainly taking the harmful side effects of the use of synthetic antibiotics into account.

### 2.2. Antifungal Activity

**Figure 2:**
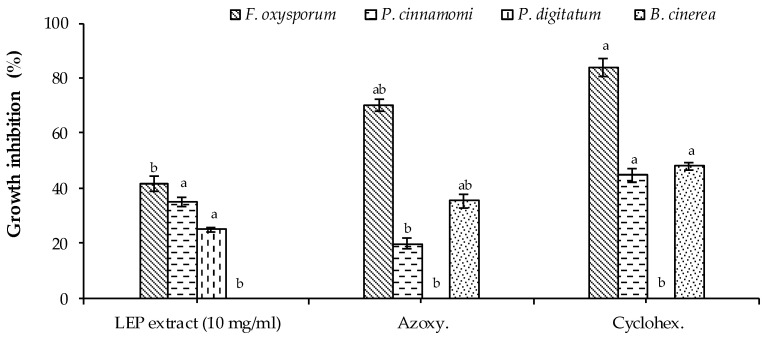
Antifungal activity of the LEP extract. Bars with different letters indicate mean values significantly different at *p* < 0.05, according to a one-way ANOVA combined with Tukey’s post-hoc test. Data are expressed as the mean of three replicates ± SD.

The results of the in vitro antifungal activity assay demonstrated that the LEP extract was less active against *F. oxysporum* and *B. cinerea* compared to the two fungicides used as a positive control (C+ve) (Figure 2), whereas the LEP extract showed the highest significant fungicidal activity against *P. cinnamomi* equal to cycloheximide (0.1 µL/mL). It was also observed that the LEP extract showed the highest significant fungicidal activity against *P. digitatum* compared to both C+ve references (Figure 2).

### 2.3. Anti-Acetylcholinesterase Activity

The LEP extract was tested for AChE activity using Ellman’s colorimetric method. The results showed a good inhibitory activity with the concentration required for achieving 50% inhibition of the enzyme (IC_50_) value of 133.0 µg/mL. Moreover, at a concentration of 250 µg/mL, the inhibition percentage (99.0% ± 4.1%) was comparable to the used control (galanthamine) (Table 1).

### 2.4. Antioxidant Activity

**Figure 3:**
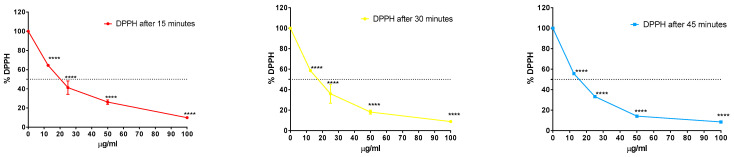
Antioxidant activity of the LEP extract. Values represent the percent of 2,2-diphenyl-1-picrylhydrazyl (DPPH) remaining after 15, 30 and 45 min. **** Values are significantly different at *p* < 0.0001, according to a two-way ANOVA combined with Tukey’s post-hoc test. Data are expressed as the mean of three replicates ± SD.

The antioxidant activity of the LEP extract was assessed by the 2,2-diphenyl-1-picrylhydrazyl (DPPH) assay. The inhibition of DPPH was dose- and time-dependent, as shown in Figure 3. The IC_50_ values ranged between 19.77 µg/mL after 15 min and 14.74 µg/mL after 45 min of exposure. These results were comparable to the antioxidant reference “vitamin C” (1.95 µg/mL after 45 min).

## 3. Discussion

The antimicrobial activity of the LEP extract confirmed the previous results [6], showing the antibacterial effects of different pomegranate peel extracts against other pathogenic bacteria such as *Listeria monocytogenes*, *Staphylococcus aureus*, *E. coli* and *Yersinia enterocolitica* both in vitro and *in situ*. Orak and coworkers [36] studied the antibacterial activity of pomegranate peel extracts against *S. aureus*, *E. coli* and *Salmonella enteritidis* and the antifungal activity against *Aspergillus parasiticus*, showing that all tested extracts showed promising antibacterial and antifungal activities against all tested bacterial and fungal strains. In addition, food-borne diseases caused by *S. aureus* and *L. monocytogenes* have been inhibited effectively by pomegranate peel methanolic extracts [15].

Furthermore, the peel extract has been successfully used in improving*Capsicum* shelf life against *Colletotrichum gloeosporioides* due to the polyphenol constituents such as punicalagin, catechin and rutin [37]. The notable antimicrobial effect of the LEP extract could be related to the high content of polyphenols—in particular, flavonoids and tannins [3,6,15,22,24]. On the other hand, the observed antimicrobial activity of the studied LEP extract may be also correlated to the polysaccharides, as reported by Miguel and coworkers [6]. The possible mechanism of the antimicrobial effects is correlated to the chemical structure of the phenolic compounds. Their lipophilic nature enables them to penetrate easily into the pathogen cell membrane and also to interfere with enzymes responsible for energy and protein production in the microorganism’s cells, hence causing cell death [38]; instead the antimicrobial activity of the polysaccharides might be related to their chemical structure and ester sulfate groups [39].

Meanwhile, functional foods are potential sources of compounds that can prevent and/or treat many diseases, such as neurodegenerative, by inhibiting acetylcholinesterase (AChE). Few previous studies have reported the anti-acetylcholinesterase activity of pomegranate peel extracts, even if their healthy and antioxidant proprieties are well-documented [13,14]. Moreover, the activity of acetylcholinesterase was measured in the cortex and hippocampus of mice, and in both cases, the treatment with a pomegranate extract promoted a significant reduction in the activity of AChE [40]. In our study, the LEP extract showed a promising activity compared to the positive control. In our study, the LEP extract showed a good activity compared to positive control. In fact, the extract, at concentration of 250 µg/mL, inhibited at 99.0% the enzyme and galanthamine reached maximum activity (inhibition of 94.2%) at the highest concentration of 10 μg/mL.

Our sample was more active than the water fraction of an ethanolic extract of pomegranate peel tested by Sakivin and coworkers [14]. In fact, at the same concentration (100 μg/mL), the ethanolic extract inhibited the enzyme by 27.9% compared to 44.2% in the case of our studied aqueous extract. However, further work is needed to identify the molecule(s) responsible for this activity.

Regarding the antioxidant activity, Elfalleh and coworkers [41] reported a half maximal effective concentration (EC_50_) value of 11.48 μg/mL for an aqueous pomegranate peel extract in agreement with our results, which proved that the choice of the aqueous extraction method is characterized by being simple and economical; therefore, it is preferred in various biological applications, especially in the pharmaceutical and food industries. On the other hand, Nagappan [42] reported that any plant species showing promising bioactivity in an aqueous extract is considered as a valuable health resource for poor communities.

Moreover, our experiments showed that the antioxidant effect was very fast, and it was seemed that there was not an influence of the reaction time on the radical scavenging activity. In fact, L-ascorbic acid also reacted very rapidly with DPPH [43], and its reaction was completed within three min, thus confirming the fast kinetic behavior of this compound. The LEP extract showed a similar behavior after 15 min; the IC_50_ value was 19.77 µg/mL. The available literature reported that different pomegranate peel extracts exhibited various degrees of antioxidant activity [44], whereas the water extract at 75 and 100 mg/mL showed a lower antioxidant activity compared to other extracts having the same effect. Thus, the waste products of pomegranate (peel and seed) with high antioxidant activity could be useful as natural preservatives in the food industry [28].

## 4. Materials and Methods

### 4.1. Chemicals

Acetylcholinesterase (AChE) type VI-S from *Electrophorus electricus* (electric eel), EC 3.1.1.7, 245 U/mg solid, 5,5′-dithiobis (2-nitrobenzoic acid) (DTNB) and acetylthiocholine iodide (AChI) were all purchased from Sigma-Aldrich (St. Louis, MO, USA). Ascorbic acid was purchased from Fluka, Buchs, Switzerland.

### 4.2. Plant Material

Pomegranate fruits (cv “Wonderful”) were collected following a randomized design in an orchard located in the province of Foggia (Apulia Region, Italy) during the seasons of summer/autumn 2018. The fruits were initially dried at 50 °C in room temperature for 48 h.

### 4.3. Extraction

The leathery exocarp peels of 3 fruits of pomegranate were sterilized using 3.0% sodium hypochlorite and then washed with sterile distilled water. The sterilized peels were left to air-dry under a laminar flow at room temperature overnight. Then, 50 g of the dried materials were suspended in 25-mL sterile distilled water and homogenized in plastic-porous bags by using a metal push rod. The suspension was agitated by using a shaker (Digital control 711D, Padova, Italy) for 24 h at room temperature, then was filtered by using a Whatman^®^ filter paper (Φ 8 cm, 75 GR/M^2^) purchased from Sigma Aldrich company (Darmstadt, Germany) and centrifuged at 8944× *g* for 10 min at 5 °C (IEC-Micromax RF, Thermo, Sparta, USA). Finally, the extract was stored at −20 °C for future use. The supernatant was membrane-filtered (0.45 μm; Minisart, Darmstadt, Germany) and concentrated under reduced pressure at 45 °C. The dried sample was resuspended in distilled water and adjusted at 10 mg/mL.

### 4.4. In Vitro Antibacterial Activity

The bactericidal activity was evaluated by the disc diffusion method [45] in King B (KB) medium against G−ve bacteria *E. coli* and *X. campestris* and G+ve ones *B. megaterium* and *C. michiganensis*. All tested bacteria were conserved in the collection of School of Agricultural, Forestry, Food and Environmental Sciences (SAFE) and re-activated on nutrient agar media at 30 ± 2 °C. Briefly, twenty µL of suspension were inoculated at the two following concentrations (10 mg/mL and 5 mg/mL), and the plates were incubated at 30 ± 2 °C for 48 h. Tetracycline and kanamycin were used as positive controls (C+ve) at 160 µg/mL and 50 µg/mL, respectively. The diameter of inhibition zone was measured. Then, the antibacterial activity was expressed as the growth inhibition percentage using the following Equation (1) [32]:

(1) $$\mathrm{Growth}\;\mathrm{inhibition}\;\left(\%\right)=100-\left[\frac{\left(Gc-Gt\right)}{Gc}\right]\times 100;$$

where *Gc* is the average diameter of bacterial grown in the control plate in mm, and *Gt* is the average diameter of inhibition zone in mm.

### 4.5. In Vitro Antifungal Activity

The tested phytopathogenic fungi were *F. oxysporum*, *P. cinnamomi*, *P. digitatum* and *B. cinerea.* The fungi were maintained as pure cultures at 4 °C in the mycotheca of SAFE and recultivated on potato dextrose agar (PDA) at 22 ± 2 °C. The LEP extract was mixed into a PDA medium at a concentration of 10 mg/mL. Then, 0.5 cm of the fungal disc were inoculated in the Petri dish and incubated at 22 °C for 96 h. Azoxystrobin at 1.0 µL/mL and cycloheximide at 0.1 µL/mL were used as C+ve. The fungitoxicity was expressed as the growth inhibition percentage following Equation (2), as proposed by Zygadlo and coworkers [46]:

(2) $$\mathrm{Growth}\;\mathrm{inhibition}\;\left(\%\right)=\left[\frac{\left(Gc-Gt\right)}{Gc}\right]\times 100;$$

where *Gc* represents the average diameter of fungal mycelium in PDA (control), and *Gt* represents the average diameter of fungal mycelium on the treated PDA dish.

### 4.6. In Vitro Anti-Acetylcholinesterase Activity

AChE inhibitory activity assay was performed according to a previously described spectrophotometric method [47] with minor modifications [48]. Briefly, in a total volume of 1 mL, 415 μL of Tris-HCl buffer 0.1 M (pH 8), 10 μL of different concentrations of extract dissolved in methanol and 25 μL of AChE solution (0.28 U/mL) were incubated for 15 min at room temperature. Seventy-five microliters of a solution of AChI (1.83 mM) and 475 μL of DTNB were added, and the final mixture was incubated for 30 min at room temperature. Absorbance was measured at 405 nm by a spectrophotometer (Thermo Scientific Multiskan GO, Monza, Italy). Galanthamine was used as the C+ve. Bi-distillated water, instead of the extract or galanthamine, was used as C−ve. The inhibition rate (%) of AChE activity was calculated by comparison with the C−ve following Equation (3):

(3) $$\mathrm{AChE}\;\mathrm{inhibition}\;\%=\left[\frac{\left(A_{0}-A_{1}\right)}{A_{0}}\right]\times 100;$$

where *A*_0_ is the absorbance of the control without the sample, and *A*_1_ is the absorbance of the sample.

### 4.7. Antioxidant Activity

Aliquots of the LEP extract were dissolved in methanol to have the final dose range from 12.5 to 100 µg/mL. The antiradical activity was determined using the sTable 1,1-diphenyl-2-picrylhydrazyl radical (DPPH) according to the Brand-Williams and coworkers method [49], with some modifications [50]. In its radical form, DPPH has an absorption peak at 517 nm, which disappears by the reduction of DPPH with antiradical compounds. For preparation of the standard curve, different concentrations of DPPH methanol solutions (10–60 µg/mL) were used. One milligram of extract was dissolved in 1 mL of DPPH solution (60 µM) to prepare the stock solution. An aliquot of MeOH solution containing different amounts of the extract was added to a DPPH solution (7.6 × 10^−5^ M), which was prepared daily and kept in the dark to have a final volume of 1 mL in a straight-sided cuvette. Methanol alone was used as the blank, whereas 1 mL of DPPH solution (60 µM) was used as the control. Absorbance at 515 nm was measured using a Thermo Scientific Multiskan GO spectrophotometer (Thermo Fischer Scientific, Vantaa, Finland) after 15, 30 and 45 min. The tested doses of the extracts were 12.5, 25, 50 and 100 µg/mL. The DPPH concentration (µg/mL) in the reaction mixture was calculated from the following calibration curve, analyzed by linear regression (r^2^: 0.9993):

Absorbance _(λ515)_ = 0.0008 + 0.0118 × [DPPH]

The scavenging capability of the tested extract was calculated using Equation (4):

(4) $$\mathrm{DPPH}\;\mathrm{scavenging}\;\mathrm{activity}\;\%=\left[\frac{\left(A_{\left(\lambda 515\right)}C-A_{\left(\lambda 515\right)}S\right)}{A_{\left(\lambda 515\right)}C}\right]\times 100;$$

where *A*_(λ515)_*C* is the absorbance of a control with no radical scavenger, and *A*_(λ515)_*S* is the absorbance of the remaining DPPH in the presence of a scavenger. The IC_50_ value was defined as the dose of the sample that reduced 50% of the initial DPPH. Ascorbic acid was used as the standard.

### 4.8. Statistical Analysis

All experiments were carried out in triplicate. Data of each experiment were expressed as the mean ± SD and statistically analyzed by two-way ANOVA using SPSS software (ver. 13, Prentice Hall, Chicago, USA, 2004), followed by Tukey’s post-hoc test, at the significance level of *p* < 0.05 or *p* < 0.0001.

## 5. Conclusions

The global interest in scientific research is to find out new substances from natural and non-synthetic sources, thus providing them for pharmaceutical manufacturing and food preservation without any harmful effects on human health. Therefore, in this research, we employed the obtained extract by using distilled water, which was an innocuous natural solvent supplied for the food industry. The findings of the current study regarding the antimicrobial, antioxidant and anti-acetylcholinesterase activities of the LEP extract, such as an, are promising for the pharmaceutical and/or food industry. In addition, the waste products of pomegranates could be suitable to be utilized as natural preservatives in the food industry because of their antimicrobial and antioxidant properties.

## Figures and Tables

**Table 1:** Anti-acetylcholinesterase activity.

Sample	Concentration (µg/mL)	AChE Inhibition Activity (%)	IC_50_ Values (µg/mL)
LEP extract	250	99.0 ± 4.1	133.0 ± 1.2
	100	44.2 ± 0.4	
	50	14.5 ± 0.8	
	25	n.a.	
Galantamine	10	94.2 ± 1.4	0.4 ± 0.2

AChE, acetylcholinesterase; LEP, leathery exocarp. n.a., not active Data are expressed as the mean of three replicates ± SD.

## Data Availability

Not applicable.
